# The Intensity of Radiotherapy-Elicited Immune Response Is Associated with Esophageal Cancer Clearance

**DOI:** 10.1155/2014/794249

**Published:** 2014-05-22

**Authors:** Jin-lu Ma, Long Jin, Yao-Dong Li, Chen-chen He, Xi-jing Guo, Rui Liu, Yun-Yi Yang, Su-xia Han

**Affiliations:** ^1^Department of Oncology, The First Affiliated Hospital of Xi'an Jiaotong University, No. 277 Yanta West Road, Xi'an 710061, China; ^2^Department of General Surgery, The Affiliated Hospital of Xi'an Medical College, No. 48 Fenghao West Road, Xi'an 710077, China

## Abstract

Radiation therapy is one of the standard therapeutic modalities for esophageal cancer, achieving its main antitumor efficacy through DNA damage. However, accumulating evidence shows that radiotherapy can substantially alter the tumor microenvironment, particularly with respect to its effects on immune cells. We hypothesized that the immune response elicited by radiotherapy may be as important as the radiation itself for successful treatment. More specifically, immunomodulatory cytokines may enhance the effectiveness of radiotherapy. To investigate this hypothesis, we measured changes in the serum interferon-gamma (IFN-**γ**) and interleukin-2 (IL-2) concentrations during radiotherapy and compared these modifications with outcomes. We found that serum concentrations of IL-2 and IFN-**γ** were positively associated with local response to radiotherapy in esophageal cancer. More generally, the intensity of the radiotherapy-elicited immune response was positively associated with local response to radiotherapy in esophageal cancer. Changes in serum IL-2 and IFN-**γ** concentrations were further associated with increased risks of acute hematologic toxicity and acute organ toxicity of the esophagus, lung, and skin. These results suggest that deciphering the mechanisms of radiotherapy-elicited immune response may help in the development of therapeutic interventions that would enhance the efficacy of radiotherapy and convert some ineffective responses to effective responses.

## 1. Introduction

Radiotherapy is one of the standard therapeutic modalities for patients with esophageal cancer. However, not all individuals respond equally to this therapy [1]. In practice, cases of esophageal cancer treated with radiotherapy can often be divided into 2 groups: those with effective responses, in which radiation can control or cure tumors, and those with ineffective responses, in which radiotherapy has little or no efficacy [2–4]. The underlying reasons for these differences in response are incompletely understood. Many studies of tissue and cellular responses to radiation have focused on the damage that is caused to proliferating malignant cells, leading to their deaths. Historically, topics of study related to radiotherapy have included the cell cycle, apoptosis, and DNA repair and survival [5, 6]. Recently, accumulating evidence has suggested that radiation also leads to significant alterations to the tumor microenvironment, particularly with respect to its effects on immune cells. It has been reported that radiotherapy can induce various tumor cell death modalities, releasing tumor-derived antigens and danger signals, either of which could trigger antitumor immune responses [7, 8].

The rate of macrophage infiltration is relatively high in tumor tissue [9]. Macrophage infiltration is present at the tumor margins and around necrotic foci. Macrophages exposed to Th1 cytokines, including interferon-gamma (IFN-*γ*) and interleukin-2 (IL-2), exhibit enhanced cytotoxic activity, production of proinflammatory cytokines, antigen presentation, and antitumor cellular immune response [10, 11]. Accordingly, IL-2 and IFN-*γ* have important roles in antitumor immunity. For example, IL-2 stimulates natural killer (NK) cell activity or cytotoxic T lymphocytes (CTLs) to kill tumor cells. IFN-*γ* is produced by the activation of T cells, and it can strengthen both the host T cell receptors' dependence and human leukocyte antigen (HLA) restriction to cytotoxic T cells, increasing surface major histocompatibility complex (MHC) antigen expression, tumor necrosis factor concentrations, antitumor angiogenesis, and other antitumor responses [12, 13]. IFN-*γ* can be controlled by regulating the Fas/FasL expression of tumor cells and enhancing the sensitivity of tumor cells to the Fas-mediated apoptosis pathway, thereby reducing the ability of tumor cells to evade the immune system attack and, accordingly, inhibiting the malignant proliferation of tumor cells [14–16].

Therefore, we hypothesized that the immune response elicited by radiotherapy may be as important as the radiation itself for successful treatment. Further, we hypothesized that immunomodulatory cytokines may enhance the effectiveness of radiotherapy. However, few studies have investigated serum IL-2 and IFN-*γ* concentrations in effective and ineffective responses to radiotherapy. Particularly, there was not enough evidence to compare the changes in these concentrations during effective and ineffective responses to radiotherapy. In the present study, we sought to provide such evidence, specifically investigating correlations between radiotherapy outcomes and changes in the serum IL-2 and IFN-*γ* concentrations during radiotherapy for esophageal cancer.

Some studies have shown that the expression of cytokines (IFN-*γ* and IL-2) is associated with radiation-related tissue damage and inflammation [17, 18]. To clarify the relationship between cytokine profiles and acute toxicity induced by radiotherapy, we also examined the associations between serum concentrations of IL-2 and IFN-*γ* and radiotherapy-related acute toxicities (hematologic or of the esophagus, lung, or skin), which were graded prospectively.

## 2. Patients and Methods

### 2.1. Patient Eligibility and Treatment

This study was approved by the Ethics Committee of The First Affiliated Hospital of Xi'an Jiaotong University (Xi'an, China), and all participating patients gave their written informed consent. The study included 63 patients diagnosed with histopathologically confirmed squamous cell carcinoma of the esophagus (cT1–4, any N, any M) at The First Affiliated Hospital of Xi'an Jiaotong University, from February 2013 to July 2013. In this study, clinical staging was performed using endoscopy, a barium swallow, computed tomography (CT) scans of the abdomen and chest, a pulmonary function test, a complete blood cell count, a serum chemistry profile, serum creatinine clearance, electrocardiography, complete history assessment and physical examination, and an assessment of Karnofsky performance status [19]. A treatment plan was then designed by a multidisciplinary tumor board. Eligible patients who were inoperable and were not fit for concurrent chemotherapy were treated with radiotherapy alone at the start of therapy. Here, inoperability was defined by the presence of distant metastases or an unfavorable preoperative risk score. Radiotherapy was administered in daily fractions of 2.0 Gy (5 times per week) with a total dose of 60–66 Gy. Linear particle accelerators (lilacs) were used with a 10 MV X-ray beam and a multiple field technique. All patients were treated using three-dimensional planning techniques.

### 2.2. Serum Collection and Enzyme-Linked Immunosorbent Assay

Blood was drawn once per week during radiotherapy; serum samples were separated by centrifugation at 3000 rpm for 5 min and stored at −80°C. Serum IL-2 and IFN-*γ* were measured with commercially available enzyme-linked immunosorbent assay kits (Westang Biotechnology Company, Shanghai, China) following the manufacturer's instructions. Samples were normalized to total protein as determined by a bicinchoninic acid assay (Westang Biotechnology Company).

### 2.3. Treatment-Related Toxicity

Acute treatment-related toxicity was evaluated weekly during radiotherapy and documented using the year 2009 National Cancer Institute's Common Toxicity Criteria (NCI-CTC, version 4.0). Supportive treatment was extensive and included pain management, dietary counseling, and leukocyte-stimulating agents administered orally or by injection, if necessary [15].

### 2.4. Efficacy Assessment

Short-term local efficacy in the esophagus was evaluated 4 weeks after finishing treatment and every 3 months for 1 year thereafter. The assessment included physical examination, history, endoscopy, a barium swallow, and thoracic/abdominal CT scans. In this study, short-term efficacy was analyzed according to the World Health Organization Response Evaluation Criteria in Solid Tumors [20]. Specifically, outcomes were classified as complete remission (CR), partial remission (PR), stable disease (SD), or progressive disease (PD), and the total effective response rate was defined as CR + PR.

### 2.5. Statistical Analysis

Fisher's exact test was used to compare categorical data. Between-group and between-sample differences in numeric data were assessed using the two-tailed Student's *t*-test. The Wilcoxon signed-rank test was used to assess the statistical significance of associations between radiation and cytokine expression, using aggregated median cytokine concentrations at baseline and during radiotherapy. The signed-rank test was also used to assess the difference between cytokine concentrations at baseline and during radiotherapy. For all analyses, *P* ≤ 0.05 was considered significant.

## 3. Results

### 3.1. Patient and Treatment Characteristics

From February 2013 to July 2013, a total of 63 patients were enrolled in this study (Table 1). Radiotherapy could result in any one of the following 2 outcomes: effective response (CR + PR) or ineffective response (SD + PD). All 63 of the patients completed treatment with radiotherapy and were followed for at least 6 months. During the short-term follow-up, the local efficacy of radiation in the esophagus was assessed using physical examination, endoscopy, barium swallow, and thoracic/abdominal CT scans. Five patients (8%) showed CR and 51 patients showed PR (81%), constituting 56 patients (89%) with an overall effective response (CR + PR). Four patients had SD (6%) and 3 patients (5%) had PD, together amounting to 7 patients (11%) with an overall ineffective response. As presented in Table 1, the characteristics of the enrolled patients (including age, sex, and disease stage) were not significantly associated with the local short-term efficacy of radiation in the esophagus.

Acute hematologic toxicity (leukopenia, anemia, and thrombocytopenia) occurred in 60 of the 63 cases (95%). Four of these 60 toxicities (6.7%) were of degrees III-IV and 56 (93.3%) were of degrees I-II. Degree I-II nausea or vomiting occurred in 35 of the 63 cases (56%). Acute organ toxicity of the esophagus, lung, or skin occurred in 61 of the 63 cases (97%). Ten of these 61 cases (16.4%) were of degrees III-IV and 51 (83.6%) were of degrees I-II. The short-term local efficacy of radiation was not significantly associated with the rate of hematologic toxicities (*P* = 0.31).

### 3.2. Intrapatient Variation in Serum Cytokine Concentrations during Radiotherapy

**Figure 1:**
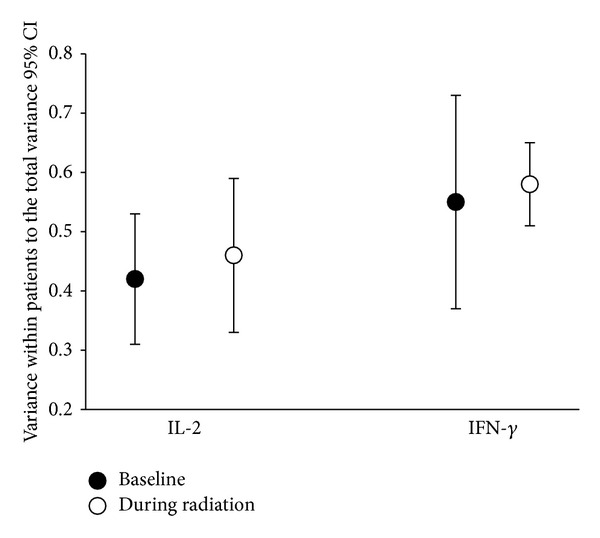
Cytokine variance within patients as a measure of heterogeneity (*n* = 63). We calculated the variance of 2 cytokines (interferon-gamma [IFN-*γ*] and interleukin-2 [IL-2]) within patients, as well as their total variances. The data points indicate the ratios of intrapatient to interpatient variances for the 2 cytokines at baseline (black circles) and during radiotherapy (white circles). The confidence interval is 95%. Based on these confidence intervals, there were no significant differences between the ratios at baseline and the ratios during radiotherapy for either of the cytokines.

Figure 1 presents the ratios of (a) the variance of the cytokine (IL-2 and IFN-*γ*) concentrations within each patient to (b) the total across-patient variance calculated for the baseline and during-radiotherapy measurements. It thereby compares intrapatient heterogeneity to interpatient heterogeneity for serum cytokine concentrations. For future studies, we suggest that this ratio of within-patient to between-patient variance should be reported as a measure of cytokine (IL-2 and IFN-*γ*) heterogeneity for the patient population. We observed that the ratios were similar at baseline and during radiotherapy for the 2 tested cytokines.

### 3.3. Changes in the Cytokine Profile Were Correlated with the Short-Term Efficacy of Radiotherapy

**Figure 2:**
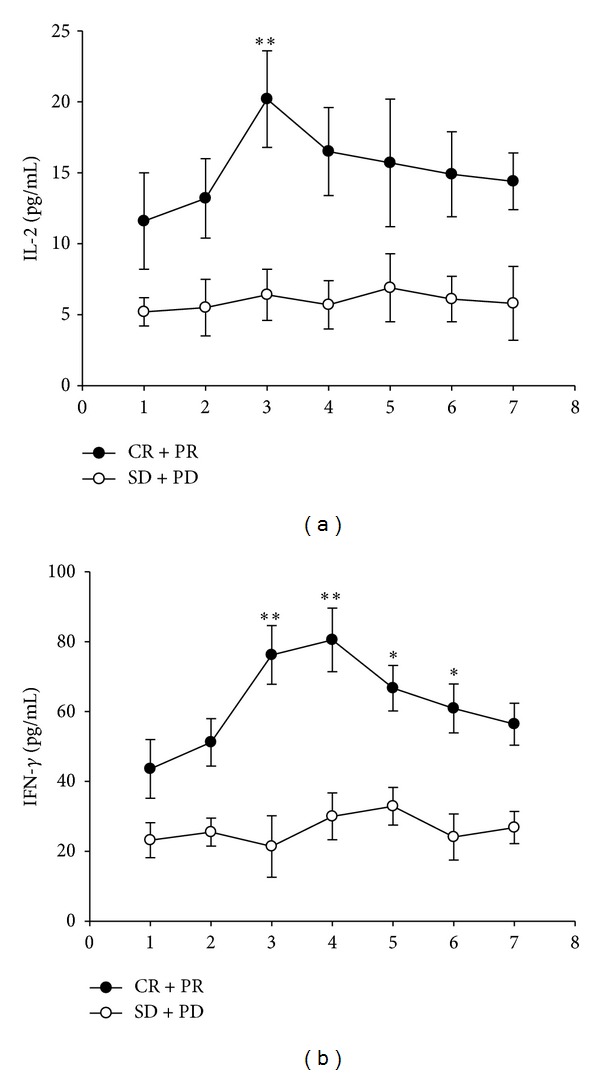
Changes in the cytokine profiles correlated with the short-term efficacy of radiotherapy. (a) Serum interleukin-2 (IL-2) comparisons for the effective response (CR + PR) and ineffective response (SD + PD) groups. (b) Serum interferon-gamma (IFN-*γ*) comparisons for the effective response (CR + PR) and ineffective response (SD + PD) groups. The total effective local response to radiotherapy relative to the serum concentrations of immunomodulatory cytokines (IL-2 and IFN-*γ*) is shown. In the effective response (CR + PR) group, serum concentrations of IL-2 and IFN-*γ* increased with the number of radiotherapy fractions that had been administered, reaching a maximum after about 2-3 weeks (10–15 fractions of radiation) and gradually decreasing thereafter. In the ineffective response (SD + PD) group, serum concentrations of IL-2 and IFN-*γ* remained approximately steady throughout the course of radiotherapy. Data are presented as mean ± 1 standard error of the mean. The asterisk indicates a statistically significant difference, as compared with the ineffective response group (Student's *t*-test, _ _*P < 0.05, _ _**P < 0.01).

Recent results have suggested that the immune system mediates many of the antitumor effects of radiotherapy [21]. Macrophages exposed to Th1 cytokines, including interferon-gamma (IFN-*γ*) and interleukin-2 (IL-2), exhibit enhanced cytotoxic activity, production of proinflammatory cytokines, antigen presentation, and antitumor cellular immune response. Therefore, IL-2 and IFN-*γ* have important roles in antitumor immune activity. We examined the serum concentrations of immunomodulatory cytokines (IL-2 and IFN-*γ*) in the effective and ineffective local response groups (Figure 2). In the effective response (CR + PR) group, serum concentrations of IL-2 and IFN-*γ* increased with the number of radiotherapy fractions that had been administered, reaching a maximum after about 2-3 weeks (10–15 fractions of radiation) and gradually decreasing thereafter. In the ineffective response (SD + PD) group, serum concentrations of IL-2 and IFN-*γ* remained approximately steady throughout the course of radiotherapy. These results indicate that the intensity of the radiotherapy-elicited immune response was positively associated with local response to radiotherapy in esophageal cancer. As shown in Figure 2, we also found that serum IL-2 and IFN-*γ* concentrations in the CR + PR group were both higher than the corresponding concentrations in the SD + PD group at the same time during radiotherapy.

### 3.4. Change in Cytokine Profile Was Correlated with Acute Toxicity Induced by Radiotherapy

**Figure 3:**
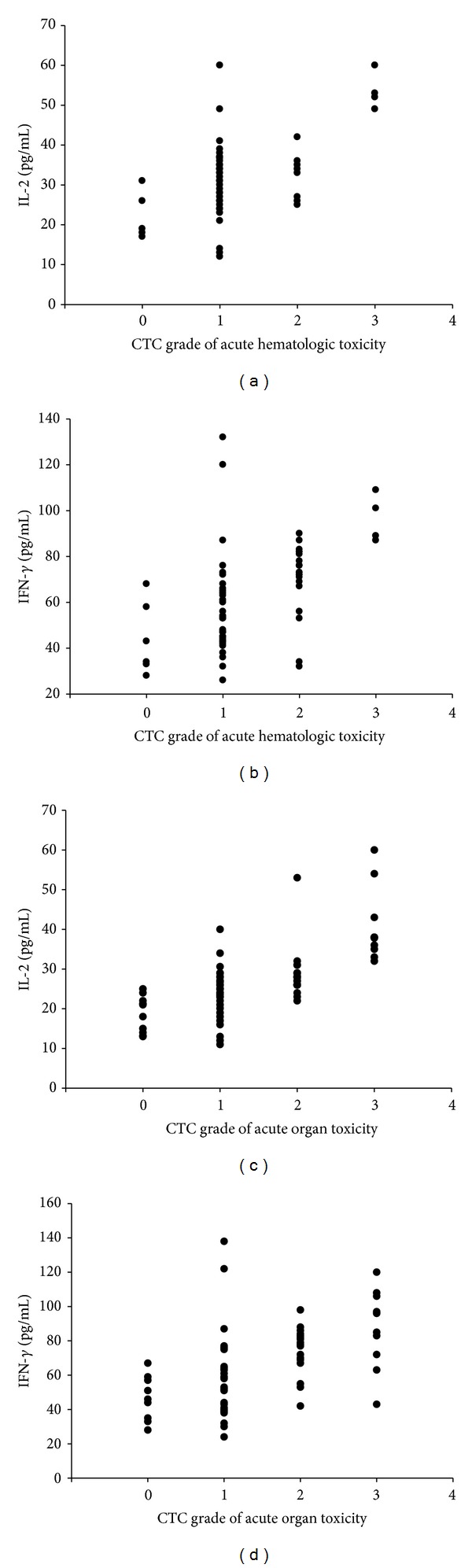
Relationship between serum concentrations of cytokines (interferon-gamma [IFN-*γ*] and interleukin-2 [IL-2]) and acute toxicity during radiotherapy. Maximum cytokine expression (IL-2 and IFN-*γ*) is presented as a function of maximum toxicity grade for the 63 patients with esophageal cancer. (a) presents the relationship between IL-2 expression and acute hematologic toxicity, (b) presents the relationship between IFN-*γ* expression and acute hematologic toxicity, (c) presents the relationship between IL-2 expression and acute organ toxicity, and (d) presents the relationship between IFN-*γ* expression and acute organ toxicity.

To clarify reported correlations between serum concentrations of IL-2 and IFN-*γ* and acute toxicity during radiotherapy, we compared the maximum cytokine concentration in each patient during radiotherapy with the maximum grade acute hematologic toxicity and acute organ toxicity (Figure 3). This comparison was performed for both IL-2 and IFN-*γ*, using separate statistical models. Our results suggest that changes in serum IL-2 and IFN-*γ* concentrations are associated with an increased probability of acute hematologic toxicity. Further, the results suggest that changes in serum IFN-*γ* concentrations are associated with an increased probability of acute organ toxicity of the esophagus and that changes in serum IFN-*γ* concentrations are associated with an increased probability of acute organ toxicity of the lung or skin.

## 4. Discussion

Radiotherapy is an important treatment modality for solid tumors. Accumulating evidence shows that radiotherapy eliminates tumor cells by changing the local tumor environment, in addition to its more direct effects. Radiation-elicited changes to local tumor microenvironment may produce inflammatory factors and enhance the antitumor immune responses locally [22]. To learn about the immune responses during cancer radiation therapy, it would be nice to detect regional lymph nodes of esophagus through immunohistochemical staining. But, in fact, it is difficult to obtain regional lymph nodes in patients during radiotherapy. Many studies have shown that IL-2 and IFN-*γ* have important roles in antitumor immune activity [23]. However, few studies have investigated serum IL-2 and IFN-*γ* concentrations in effective and ineffective responses to radiotherapy. Particularly, there was not enough evidence to compare the changes in these concentrations during effective and ineffective responses to radiotherapy. In the present study, we have provided such evidence, specifically detailing correlations between radiotherapy outcomes and changes in the serum IL-2 and IFN-*γ* concentrations during radiotherapy for esophageal cancer.

The present study enrolled 63 patients with esophageal cancer. All patients completed treatment with radiotherapy and were followed for at least 6 months. During the short-term follow-up, the local efficacy of radiation was assessed with physical examination, endoscopy, barium swallow, and thoracic/abdominal CT scans. Five patients (8%) exhibited CR and 51 patients exhibited PR (81%), constituting 56 patients (89%) with an overall effective response (CR + PR). Four patients had SD (6%) and 3 patients (5%) had PD, together amounting to 7 patients (11%) with an overall ineffective response. We found significant differences between patients with and without effective local response in terms of serum IL-2 and IFN-*γ* concentrations. In the effective response (CR + PR) group, serum concentrations of IL-2 and IFN-*γ* increased with the number of radiotherapy fractions that had been administered, reaching a maximum after about 2-3 weeks (10–15 fractions of radiation) and gradually decreasing thereafter. In the ineffective response (SD + PD) group, serum concentrations of IL-2 and IFN-*γ* remained approximately steady throughout the course of radiotherapy. Consistent with our hypothesis, results indicate that the intensity of the radiotherapy-elicited immune response was positively associated with the local response to radiotherapy in esophageal cancer.

Several studies have shown that cytokine (IFN-*γ* and IL-2) expression is associated with radiation-related tissue damage and inflammation. Increasing IL-2 and IFN-*γ* expressions were associated with an increased probability of acute toxicity induced by radiotherapy [17]. To examine the relationships between acute toxicity induced by radiotherapy and cytokine profiles, we examined associations between radiotherapy-related acute toxicities, which were graded prospectively, and serum IL-2 and IFN-*γ* concentrations. To clarify reported correlations between serum concentrations of IL-2 and IFN-*γ* and acute toxicity during radiotherapy, we compared the maximum cytokine concentration in each patient during radiotherapy with the maximum grade acute hematologic toxicity and acute organ toxicity (Figure 3). This comparison was performed for both IL-2 and IFN-*γ*, using separate statistical models. Our results suggest that changes in serum IL-2 and IFN-*γ* concentrations are associated with an increased probability of acute hematologic toxicity. Further, the results suggest that changes in serum IFN-*γ* concentrations are associated with an increased probability of acute organ toxicity of the esophagus and that changes in serum IFN-*γ* concentrations are associated with an increased probability of acute organ toxicity of the lung or skin.

In the current study, we demonstrated that serum concentrations of IL-2 and IFN-*γ* were positively associated with local response to radiotherapy in esophageal cancer. These findings suggest that the intensity of the radiotherapy-elicited immune response is positively associated with local response to radiotherapy in esophageal cancer. Further, changes in the IL-2 and IFN-*γ* serum concentrations were associated with increased probabilities of acute hematologic toxicity and acute organ toxicity of the esophagus, lung, and skin. In fact, serum levels of IL-2 and IFN-*γ* have been found positively associated with patients' outcome [24]. IL-2 and IFN-*γ* have been used in immunotherapy in some tumors like malignant melanoma [25]. But the relationship between radiotherapy inducing acute toxicity and outcome is unclear. In our future research we will research the relationships of IL-2, IFN-*γ*, and other cytokines with radiotherapy acute phase response and outcome. These results suggest that deciphering the mechanisms of radiotherapy-elicited immune response may allow the development of therapeutic interventions that would enhance the efficacy of radiotherapy and convert some ineffective responses to effective responses.

## Figures and Tables

**Table 1:** Patient and treatment characteristics related to local short-term efficacy of radiotherapy for esophageal cancer.

Study population	CR + PR	SD + PD	*P* value
*N* = 63	*N* = 56	*N* = 7	
Age (years)			
Median	65	63	0.23
Range	43–82	55–67	
Sex			
Female	19	1	0.42
Male	37	6	
Stage			
I-II	6	1	0.43
III	48	5	
IV	2	1	
Tumor length			
<5 cm	15	3	0.34
≥5 cm	4	4	
Acute hematologic toxicity			
0/I-II	53	6	0.38
III-IV	3	1	
Acute organ toxicity			
0/I-II	48	5	0.31
III-IV	8	2	

CR + PR: patients with complete or partial response; SD + PD: patients with stable disease or progressive disease.
